# Midterm Echocardiographic Outcomes of Minimally Invasive Mitral Valve Surgery in Patients With Previous Cardiac Surgery

**DOI:** 10.1016/j.atssr.2025.11.007

**Published:** 2025-12-05

**Authors:** Nicolas Mourad, Durr Al-Hakim, Rosalind Groenewoud, Richard C. Cook

**Affiliations:** 1 Faculty of Medicine, University of British Columbia, Vancouver, British Columbia, Canada; 2 Division of Cardiac Surgery, Vancouver General Hospital, University of British Columbia, Vancouver, British Columbia, Canada

## Abstract

**Background:**

Minimally invasive mitral valve repair (MVr) and mitral valve replacement (MVR) after previous sternotomy are relatively uncommon. This study reports midterm outcomes at a single institution.

**Methods:**

All patients with a history of previous cardiac surgery who underwent minimally invasive MVr and MVR with hypothermic fibrillatory arrest at our institution (Vancouver General Hospital, University of British Columbia, Vancouver, BC, Canada) between 2006 and 2024 were included. Follow-up echocardiographic reports were reviewed at 1 year, 1 to 3 years, 3 to 5 years, and 5+ years. Primary outcomes included postoperative complications, all-cause mortality, and rates of grade 3 to 4 mitral regurgitation at follow-up.

**Results:**

A total of 31 patients met the inclusion criteria (25.8% female patients), and their median age was 64 years. A total of 18 patients underwent MVR, and 13 underwent MVr. The most common previous cardiac operations were aortic valve replacement (AVR), coronary artery bypass graft, and MVR. All redo procedures were completed using hypothermic fibrillatory arrest (mean, 23.3 [2.5] °C; median duration, 143.0 minutes [interquartile range, 110.5-175.0 minutes]) for myocardial protection. The 30-day, 5-year, and 10-year all-cause mean mortality rates were 0.0% (0.0%), 12.9% (6.0%), and 25.8% (7.9%), respectively. Patients with previous AVR with or without ascending aortic replacement represented 50% of total deaths. Overall, 4 (12.9%) patients had recurrent grade 3 to 4 mitral regurgitation: 3 died, and 1 had subsequent double-redo MVR.

**Conclusions:**

Our study demonstrates low perioperative mortality rates, thus implying the safety of fibrillatory arrest for myocardial protection in redo minimally invasive MVr and MVR. We also identified that patients with AVR with or without ascending aortic replacement face a higher midterm risk, given the technical difficulty of operating with fibrillatory arrest on the anterolateral portion of the mitral valve when the aortic annulus or root is inflexible.

**In Short**

▪ Fibrillatory arrest is safe for myocardial protection in redo MIS MVr and MVR.
▪ Patients with previous AVR with or without AAR face a higher risk of poor midterm outcomes.

The minimally invasive surgery (MIS) approach in mitral valve surgery (MVS) has emerged as a promising alternative to traditional surgical approaches, especially in redo cases, where the risks of major bleeding, potential injury to vital mediastinal structures, and poor access resulting from dense adhesions are greater.^1^ In particular, patients with previous coronary artery bypass grafts (CABGs), aortic valve replacement (AVR), or calcified aortas have a higher risk of graft injuries, hemorrhage, and complex valve exposure for all redo procedures.^2^ Reoperative MVS has become increasingly common because of the more frequent implantation of bioprosthetic valves, the steady rise in prosthetic valve endocarditis, and an aging population.^3^

Despite the growing body of literature on midterm and long-term echocardiographic outcomes after an MIS approach to MVS, there remains a gap in data addressing patients undergoing redo procedures. Existing studies often focus on perioperative outcomes and mortality rates but not the midterm and long-term echocardiographic characteristics in patients undergoing redo procedures.^4^^,^^5^ This paper aims to characterize midterm echocardiographic outcomes in patients who have undergone an MIS approach to MVS after previous cardiac surgery (redo MIS MVS).

## Patients and Methods

### Study Cohort

A retrospective chart review of a total of 31 consecutive adult patients who underwent an MIS mitral valve repair (MVr) or mitral valve replacement (MVR) under hypothermic fibrilatory arrest (HFA) after previous cardiac surgery at our institution (Vancouver General Hospital, University of British Columbia, Vancouver, BC, Canada) from March 2006 to May 2024 was completed. The study was approved by the University of British Columbia Clinical Research Ethics Board. All procedures were performed by a single surgeon (R.C.). Decision making about patient selection and procedural details for MIS MVr and MVR are available in the Supplemental Materials.

### Outcomes

The primary outcomes of the study were postoperative complications, all-cause mortality, and MVr or MVR success, defined by grade 0 to 1 mitral regurgitation (MR) on follow-up transthoracic echocardiography (TTE). Secondary outcomes included intraoperative complications (eg, conversion to sternotomy), operative times, hospital length of stay, and postoperative left ventricular ejection fraction (LVEF). Postoperative complications included major wound infection, acute renal failure, stroke, postoperative atrial fibrillation, and reoperation for bleeding.

### Statistical Analysis

All data are represented as frequency (percentage) for categorical variables. Continuous variables are presented as mean (SD) if they followed a normal distribution and median and interquartile range (IQR) for nonnormal distributions. Differences in operative, postoperative, and follow-up TTE parameters were analyzed using either a 1-way repeated measures analysis of variance, Wilcoxon signed rank test, or Student *t* test. A Kaplan-Meier survival graph was created for long-term mortality. All analyses were conducted using SPSS software version 28.0.0.1 (SPSS Statistics, IBM Corp) and R software version 4.4.1 (R Foundation for Statistical Computing). Statistical significance was defined as a *P* value <.05.

## Results

Between March 2006 and May 2024, of 82 patients undergoing mitral valve reoperations, 49 (59.8%) underwent a redo sternotomy, and 33 (40.2%) patients underwent a minithoracotomy. Overall, 31 (37.8%) patients met the inclusion criteria, with 18 (58.1%) undergoing MIS MVR and 13 (41.9%) undergoing MIS MVr. Baseline clinical characteristics are summarized in Supplemental Table 1. Median age at the reoperative MIS MVS was 64 years (IQR, 55-74 years), and 8 (25.8%) patients were female. The most common previous cardiac operations were AVR (48.4%), CABG (41.9%), and MVR or MVr (19.4%). Of the 13 patients who had a previous CABG, 11 (84.6%) had a graft to the right coronary artery. A total of 4 (12.9%) patients had more than 1 previous sternotomy.

Preoperative echocardiographic data and operative details are summarized in Supplemental Table 2. All procedures were completed using fibrillatory arrest with systemic hypothermia for myocardial protection, with a mean initiation temperature of 23.3 (2.5) °C and a maintenance temperature of 26.8 (2.2) °C throughout most of the procedure. The median duration of HFA was 143.0 minutes (IQR, 110.5-175.0 minutes). The median operating room time was 386.0 minutes (IQR, 336.0-449.0 minutes), and the median cardiopulmonary bypass time was 240.0 minutes (IQR, 217.3-319.5 minutes). No patients required conversion to sternotomy. Patients with a previous AVR with or without ascending aortic replacement (AAR) had a longer average HFA duration compared with the group of patients who did not (169.5 [17.1] minutes vs 126.0 [11.1] minutes, respectively; *P* = .048).

Perioperative outcomes are summarized in Supplemental Table 3. The average hospital length of stay was 9.0 (6.2) days. Postoperatively, no patients had a stroke. The 30-day, 5-year, and 10-year all-cause mortality rates were 0.0% (0.0%), 12.9% (6.0%), and 25.8% (7.9%), respectively (Figure). Patients with a previous AVR with or without AAR represented 50.0% of the total deaths. Of the 8 patients who died, 3 (37.5%) underwent MIS MVr and 5 (62.5%) underwent MIS MVR. Compared with the rest of the cohort, the patients who died were older (75.4 [7.5] years vs 60.4 [14.4] years; *P* < .001), had more hypertension (87.5% [3.5%] vs 52.2% [5.1%]; *P* = .045), and had a slightly lower preoperative LVEF (47.5% [10.4%] vs 52.6% [9.4%]; *P* = .25).

**Figure.**
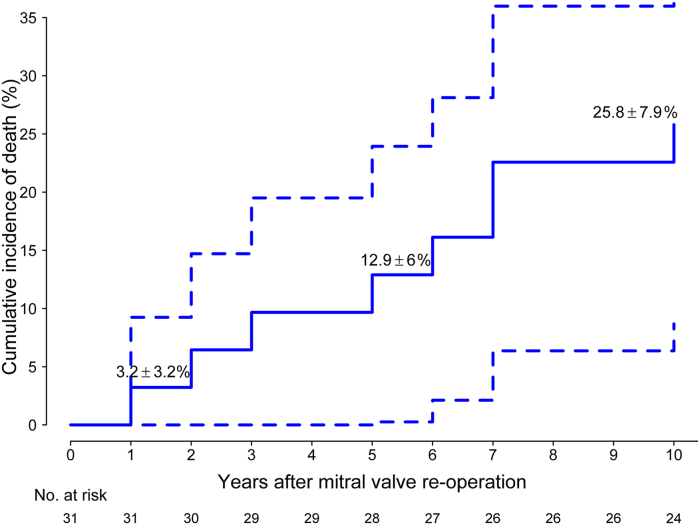
Cumulative rate curve of outcome: all-cause mortality rates over a 10-year follow-up period. Dashed lines are 95% confidence limits.

Follow-up TTE data at 5+ years were available for 20 of 31 (64.5%) patients because 7 patients were not yet 5+ years out and 3 had already died. There were significant changes of means and percentages of MR grade across the 5 follow-up time periods (*P* < .001 and *P* < .001, respectively) (Table). Overall, 4 (12.9%) patients had recurrent grade 3 to 4 MR postoperatively. Of these 4 patients, 2 patients had MVRs, and 2 patients had had previous AVR with AR. Of the 2 patients who underwent redo MIS MVR, 1 patient had a moderate to severe perivalvular leak requiring percutaneous intervention. This patient had had previous AVR with AAR. The other patient had undergone previous MVr, and this patient had a failed bioprosthetic valve with no perivalvular leak. Of the 2 patients who had failed redo MIS MVr procedures, 1 patient had undergone previous AVR with AAR. This patient had MR secondary to tethering of the posterior leaflet. Because of poor exposure of the mitral valve, it was believed that MVR would be too difficult, and therefore the valve was repaired using a downsized annuloplasty ring. The second patient who had a failed redo MIS MVr had had a previous CABG and had “P2 flail with a small and tethered anterior leaflet.” The valve was repaired with Gore-Tex neochordae (W.L. Gore & Associates, Inc) to P2 and a 32-mm Carpentier Edwards Physio II annuloplasty ring (Edwards Lifesciences), which required further downsizing to a 30-mm ring. Three of the patients (75.0%) died of worsening congestive heart failure, and 1 (25.0%) required a double-redo MVR.

**Table.** Midterm Follow-Up Echocardiographic Left Ventricular Ejection Fraction and Mitral Regurgitation Data

Variables	Preoperative (n = 31)	Within 1 y (n = 29)	1-3 y (n = 24)	3-5 y (n = 17)	5+ y (n = 20)	*P* Value
LVEF, %	51.3 [9.7]	53.4 [12.1]	51.7 [12.5]	53.4 [9.7]	51.5 [10.4]	.68
LVEF grouped						.10
LVEF ≤60%	27 (87.1)	22 (75.9)	22 (91.7)	14 (82.4)	18 (90.0)	
LVEF >60%	4 (12.9)	7 (24.1)	2 (8.3)	3 (17.6)	2 (10.0)	
MR grade	3.5 [0.9]	1.0 [0.5]	1.1 [0.8]	1.2 [0.9]	1.2 [1.1]	<.001
MR grade grouped						<.001
None (0)	1 (3.2)	5 (17.2)	4 (16.7)	2 (11.8)	5 (25.0)	
Trivial to mild (1)	0 (0.0)	20 (69.0)	16 (66.7)	13 (76.5)	10 (50.0)	
Moderate (2)	1 (3.2)	4 (13.8)	1 (4.2)	0 (0.0)	4 (20.0)	
Moderate to severe (3)	9 (29.0)	0 (0.0)	3 (12.5)	1 (5.9)	0 (0.0)	
Severe (4)	20 (64.5)	0 (0.0)	0 (0.0)	1 (5.9)	1 (5.0)	
Mitral valve gradient, mm Hg	…	6.3 [2.5]	5.2 [2.2]	6.2	5 [2.2]	…

Values are mean [SD] or n (%).

LVEF, left ventricular ejection fraction; MR, mitral regurgitation.

There was no significant change of means or LVEF percentages grouped by ≤60% or >60% across the 5 follow-up time periods (*P* = .26). Results of a Wilcoxon signed rank test showed no significant difference between preoperative and early postoperative LVEF, with 4 patients having a reduced LVEF immediately postoperatively (51.3% [9.7%] vs 53.4% [12.1%]; *P* = .48). Average LVEF remained the same preoperatively and at 5+ years of follow-up (51.3% [9.7%] vs 51.5% [10.4%]; *P* = .91).

## Comment

Performing redo cardiac surgery through a right minithoracotomy approach may allow for less lysis of pericardial adhesions than is required when approaching the mitral valve through a redo sternotomy approach, and it may therefore cause less of an inflammatory reaction. It has been the experience of the surgeon in this study that adhesions around the posterior and lateral aspects of the heart are less dense than those encountered more anteriorly.

However, dissection of adhesions around the aortic root can be particularly difficult through a small right minithoracotomy incision, thus posing a significant challenge to safe aortic clamping and delivery of antegrade cardioplegia into the ascending aorta, especially in patients with patent bypass grafts. HFA is an alternative cardioprotective method that allows continuous perfusion of the heart and avoids the need for aortic clamping, cardioplegia, and dissection around the aortic root.^6^^,^^7^ In our study, all procedures were completed using HFA with systemic hypothermia initiated at 23.3 (2.5) °C and maintained at 26.8 (2.2) °C. Previous studies of HFA reported 30-day mortality rates ranging from 0% to 7.4%,^7^, ^8^, ^9^ with 3- and 5-year survival of 90% to 91.6%,^6^ as well as a notably low rate of postoperative complications such as renal failure (1.5%) in patients with CABGs.^7^ However, previous MIS MVS studies identified an increased risk of stroke associated with HFA and reported a 3% incidence of stroke.^7^

Our 30-day all-cause mortality rate of 0% is lower than in other reports. In addition, we also observed a perioperative stroke rate of 0%, which is also lower than previously reported experiences. Long-term cardiac function was preserved in our cohort, as evidenced by the stability of baseline ejection fraction throughout the follow-up period. It is our belief that the technical factors described in the Supplemental Material are responsible for these outcomes. In particular, positioning the bed in the Trendelenburg position while the left atrium is open, simultaneously maintaining the arterial perfusion pressure higher than 80 mm Hg to keep the aortic valve closed, and infusing carbon dioxide directly into the left atrium all likely contributed to minimizing embolism of air or other materials to the cerebral vessels. Deairing of the left side of the heart can be challenging in redo procedures performed through a right minithoracotomy incision, particularly when there is no aortic root vent in position. Therefore, we believe that preventing accumulation of air in the left atrium and ventricle by infusing carbon dioxide directly into the left atrium is particularly important. We also believe that using hypothermia to induce fibrillatory arrest, as opposed to using an electrical fibrillatory approach, has the supplemental benefit of providing an additional layer of myocardial protection, which may have contributed to the low perioperative mortality rate.

At midterm follow-up, we observed that 4 patients (12.9%) had moderate to severe or severe MR. Two of the patients (50%) had had undergone previous AVR with AAR, which we believe reflects the technical difficulty of performing these operations on patients who have previously had AVR with or without AAR. Reoperation on the mitral valve in these patients can be particularly challenging given the rigid aortic annulus or aortic root.^10^ This can hinder visualization and manipulation of the mitral valve tissue in the area of the anterolateral trigone.^10^ This issue is further demonstrated in the longer mean HFA duration in these patients, compared with patients who did not have a history of previous AVR with or without AAR. Furthermore, 50% of patients who had grade 3 to 4 MR recurrence and 50% of all late deaths were observed in those patients with a previous history of AVR with or without AAR. Because of these observations, we have stopped performing redo MIS MV surgery with HFA in patients who have had a previous AVR with or without AAR. An established alternate approach for these patients would be a redo double-valve replacement through sternotomy.

### Study limitations

The retrospective, nonrandomized design of this single-center, single-surgeon study introduces biases such as selection bias, and the small sample size limits the generalizability of the findings. The lack of a comparison group limits outcome comparisons between the 2 approaches. Additionally, variable follow-up periods and limited echocardiographic data for recent redo operations may affect the results. Only echocardiography reports available in the electronic medical system were included, thus potentially missing data from imaging performed elsewhere.

### Conclusion

Our study demonstrates low perioperative mortality rates, thereby implying the safety of HFA for myocardial protection in MIS MVr and MVR after previous cardiac surgery. We also observed that patients with a history of previous AVR or aortic root procedures had longer HFA times and poorer midterm outcomes, findings suggestive of added technical complexity in this patient population.
