# Supplementary material for: Midterm Echocardiographic Outcomes of Minimally Invasive Mitral Valve Surgery in Patients With Previous Cardiac Surgery
Source: Ann Thorac Surg Short Rep. 2025 Dec 5;4(2):585–9. doi: 10.1016/j.atssr.2025.11.007 (PMC13245334; doi:10.1016/j.atssr.2025.11.007)
Supplement: Supplementary Material [file mmc1.docx]

**Supplemental Material**

| Contents | Page |
| --- | --- |
| Additional Decision-Making and Surgical Technical Information | 2-3 |
| Follow-up | 4 |
| Supplemental Table 1. Patient characteristics at baseline | 5-6 |
| Supplemental Table 2. Preoperative echocardiographic data and operative details | 7-8 |
| Supplemental Table 3. Perioperative outcomes | 9 |
| References | 10 |

*Decision-Making and Surgical Technique*

All patients underwent intraoperative transesophageal echocardiogram (TEE). If a repair was performed, a pre-discharge transthoracic echocardiogram (TTE) was performed. Procedures included MIS redo mitral valve repair (MVr) or replacement (MVR), with or without concomitant procedures such as tricuspid valve surgery, atrial septal defect repair, or patent foramen ovale closure. Patients were accepted for redo MIS MVS unless the following contraindications were present: (1) very severe pulmonary hypertension (>45 mmHg), (2) severe lung disease precluding single lung ventilation, (3) very low LVEF (<30%), (4) moderate or severe aortic regurgitation, (5) severe pleural adhesions, (6) severe peripheral vascular disease, (7) younger patients (< 50 years old) with Barlow’s type valve with anticipated complex repair, and (8) previous right thoracotomy. In addition to these, following the publication of a trial by Whitlock et al.^1^ that demonstrated a benefit to left atrial appendage ligation to minimize risk of ischemic stroke, patients with atrial fibrillation have been excluded from MIS MVS. Also, patients with an intrathoracic anteroposterior diameter of under 10cm or pectus excavatum were excluded due to local observation of a higher rate of conversion to sternotomy and failed repairs observed in patients with these anatomic features.

After opening the left atrium through the interatrial groove, a left atrial retractor was placed, and the valve was inspected. An “Endocameleon” scope (Endocameleon Hopkins Telescope, Tuttlingen, Germany: Karl Storz) was placed into the right pleural space via a 10mm port placed 1-2 interspaces below the surgical incision and was used to aid in visualization of the valve. However, most of the surgery was performed using direct vision. In cases of MVr, the repair was completed using a variety of strategies, including leaflet resection, cleft closure, leaflet and annulus decalcification, and/or neochord placement. An Edwards Physio II annuloplasty ring placed in all cases, except for one. In cases of MVR, St Jude Epic porcine valves were implanted.

TEE was performed for all patients, and used to guide de-airing, assess valvular and ventricular function and to rule out perivalvular leak. The pericardium was then closed using 1-2 interrupted vicryl sutures, and a single 28fr angled chest tube was placed using the scope port incision.

All patients were transferred to the cardiac intensive care unit while intubated. No patients required mechanical support to separate from cardiopulmonary bypass.

*Follow-up*

Patients underwent outpatient follow-up appointments where TTE’s were performed. When available, we collected one per year until the end of the follow-up period. The follow-up period was concluded on May 31, 2025.

**Supplemental Table 1.** Patient characteristics at baseline

| Variables | Summary (total n=31) |
| --- | --- |
| Age in years at previous cardiac operation, median (IQR) | 58.0 (46.0-64.0) |
| Age in years at re-operative MIS MVS, median (IQR) | 64.0 (55.0-74.5) |
| Male gender, n (%) | 23 (74.2) |
| BMI, mean (SD) | 26.5 (± 4.3) |
| EF%, mean (SD) | 51.3 (± 9.7) |
| Dialysis, n (%) | 0 (0.0) |
| Hypertension, n (%) | 19 (61.3) |
| Endocarditis, n (%) | 2 (6.5) |
| Lung disease, n (%) | 8 (25.8) |
| Peripheral vascular disease, n (%) | 1 (3.2) |
| Previous cerebrovascular accident, n (%) | 2 (6.5) |
| Previous cardiac operations, n (%) |  |
| AVR | 15 (48.4) |
| CABG | 13 (41.9) |
| MVR/r | 6 (19.4) |
| Aortic surgery | 5 (16.1) |
| ASD repair | 3 (9.7) |
| Other: TV repair, Septal Myectomy, PDA closure, LAA closure | 4 (12.9) |
| Previous PCI, n (%) | 4 (12.9) |
| Previous MI, n (%) | 7 (22.6) |
| Angina, n (%) | 9 (29.0) |
| Heart failure, n (%)  Type I  Type II  Type III  Type IV | 31 (100.0)  1 (3.2)  9 (29.0)  18 (58.2)  3 (9.7) |
| Atrial fibrillation, n (%) | 17 (54.8) |

EF = ejection fraction; AVR=aortic valve replacement; CABG=coronary artery bypass graft; MIS = minimally invasive; MVR = mitral valve replacement; MVS = mitral valve surgery; ASD = atrial septal defect; TV = tricuspid valve; PDA = patent ductus arteriosus; LAA = left atrial appendage; PCI = percutaneous coronary intervention; MI = myocardial infarction

**Supplemental Table 2.** Preoperative echocardiographic data and operative details

| Variables | Summary (total n=31) |
| --- | --- |
| Aortic stenosis, n (%) | 4 (12.9) |
| Mitral stenosis, n (%) | 3 (9.7) |
| Aortic insufficiency, n (%) | 6 (19.4) |
| Mitral regurgitation, n (%) | 30 (96.8) |
| Tricuspid regurgitation, n (%) | 29 (93.5) |
| Procedure status, n (%)  Elective  Urgent | 25 (80.6)  6 (19.4) |
| Conversion to sternotomy, n (%) | 0 (0.0) |
| Indication for Procedure, n (%) |  |
| Symptomatic grade 2-4 MR | 29 (93.5) |
| Infective Endocarditis | 2 (6.5) |
| Intraoperative evaluation, n (%) |  |
| Heavily abnormal valve | 4 (12.9) |
| Heavily calcified valve | 5 (16.1) |
| Replacement, n (%) | 18 (58.1) |
| St. Jude Epic | 14 (77.8) |
| Mechanical | 4 (22.2) |
| Repair, n (%) | 13 (41.9) |
| Neochord use | 2 (15.4) |
| Isolated P2 repair | 1 (7.7) |
| Anterior leaflet repair | 6 (46.2) |
| Commissure repair | 4 (30.7) |
| No leaflet repair | 4 (30.7) |
| Ring annuloplasty |  |
| CE Physio II | 12 (92.3) |
| No ring | 1 (8.7) |
| Operation duration in minutes, median (IQR) | 386.0 (336.0-449.0) |
| CPB duration in minutes, median (IQR) | 240.0 (217.3-319.5) |
| HFA duration in minutes, median (IQR) | 143.0 (110.5-175.0) |

MR=Mitral regurgitation, CPB=cardiopulmonary bypass, HFA=hypothermic fibrillatory arrest

**Supplemental Table 3.** Perioperative outcomes

| Variables | Summary (total n=31) |
| --- | --- |
| Reoperation for Bleeding, n (%) | 3 (9.7) |
| Acute Kidney Injury, n (%) | 3 (9.7) |
| Post-Operative Atrial Fibrillation, n (%) | 2 (6.5) |
| Major Wound Infection, n (%) | 0 (0.0) |
| Cerebrovascular Accidents, n (%) | 0 (0.0) |
| Hospital Length of Stay in days, mean (SD) | 9.0 (± 6.2) |
| 30-day mortality, n (%) | 0 (0.0) |
| Total mortality, n | 8 |

**References**

1. Whitlock RP, Belley-Cote EP, Paparella D, et al. Left atrial appendage occlusion during cardiac surgery to prevent stroke. *N Engl J Med*. 2021;384(22):2081-2091.
